# Avoiding the danger that stop smoking services may exacerbate health inequalities: building equity into performance assessment

**DOI:** 10.1186/1471-2458-7-198

**Published:** 2007-08-09

**Authors:** Allan Low, Louise Unsworth, Anne Low, Iain Miller

**Affiliations:** 1 Freelance health economist, Wales, UK; 2 County Durham and Darlington Public Health Team, County Durham Primary Care Trust, Durham, UK; 3 Director of Public Health (Retired), Wales, UK

## Abstract

**Background:**

The UK is the only developed country to have established a nation-wide stop smoking treatment service. Apart from addressing tobacco dependence, which is the leading preventable cause of ill health and premature death, smoking cessation has been identified by the UK department of health as a service priority for reducing gaps in health between disadvantaged groups and the country as a whole. However smoking cessation tends to be more successful among affluent than disadvantaged groups. This means that for stop smoking services there is a trade-off to be had in terms of maximising the number of quitters and reducing socioeconomic inequalities in smoking prevalence. Current performance targets for the national stop smoking services in the UK are set only in terms of numbers of quitters, which does not encourage the adoption of strategies to reduce socioeconomic inequalities in smoking prevalence.

**Discussion:**

This paper proposes an assessment framework, which allows the two dimensions of overall reduction in smoking prevalence and reductions of inequalities in smoking prevalence to be assessed together. The framework is used to assess the performance over time of a stop smoking service in Derwentside, a former Primary Care Trust in the North East of England, both in terms of meeting targets for the overall number of quitters and in terms of reducing socioeconomic inequalities in smoking prevalence.

The example demonstrates how the proposed assessment framework can be applied in practice given existing records kept by stop smoking services in England and the available information on smoking prevalence at small area level. For Derwentside it is shown that although service expansion was successful in increasing the overall number of quitters, the service continued to exacerbate inequality in smoking prevalence between deprived and affluent wards.

**Summary:**

The Secretary of State for Health in the UK has warned about the dangers of health promotion services and messages being taken up more readily by the better-off, thus exacerbating health inequalities. Because smokers from affluent backgrounds are more successful at quitting than those living in deprived circumstances, it is important to build an equity element into the monitoring of individual stop smoking services. Otherwise the danger highlighted by the Secretary of State for Health will go undetected and unaddressed.

## Background

In the UK, as in many developed countries, tobacco use is the single, greatest cause of preventable illness and premature death [1]. Most heath systems in these countries incorporate stop smoking programmes in one way or the other. For example in the USA smoking cessation clinics have been established by community pharmacists [2]. In some states of the USA Medicaid includes coverage for treatment of tobacco dependence [3]. National programmes have aimed to improve treatments being offered through the establishment of smoking cessation pathways (e.g Canada [4]) or guidelines (e.g. France [5]). The UK is the only country to have introduced a national stop smoking treatment service. There are a number of aspects of this experience that may be of relevance to other countries [6]. One aspect, which is the subject of this paper, is the way in which the stop smoking services in England are evaluated for their impact on smoking prevalence as a whole, as well as on socioeconomic inequalities in smoking prevalence.

Smoking cessation tends to be less successful among more disadvantaged groups where smoking prevalence is highest [7-11]. The NHS Stop Smoking Service (SSS) in England originally targeted socially disadvantaged groups by establishing support and treatment services for those wishing to stop smoking in deprived localities known as Health Action Zones. The health inequalities reduction credentials of this targeted provision are clear.

The service has since been rolled out to all Primary Care Trusts (PCTs) in the country. Under this universal provision there remains the expectation that SSS will contribute to reducing health inequalities. For example, in the NHS Operating Framework for 2006/07, the Department of Health identifies smoking cessation as a service priority that will make the most progress in reducing health inequalities [12].

The expectation that universal provision of SSS will contribute to reducing health inequalities is problematic when it is known that quit rates by smokers from disadvantaged socioeconomic groups are lower than those from affluent groups [9-11,13]. The Secretary of State for Health has warned about the dangers of health promotion services and messages being taken up more readily by the better-off, thus exacerbating health inequalities [14].

The danger that SSS could exacerbate health inequalities is compounded by the current performance assessment of stop smoking services. Targets are set and monitored only in terms of the total numbers of quitters in each SSS [15]. No assessment is made of how quitters are distributed across socioeconomic groups.

This paper proposes a framework, which allows an assessment of the contribution of a SSS to reducing overall smoking prevalence (number of quitters per smoker) to be combined with an assessment of the contribution of a SSS to reducing inequalities in smoking prevalence. The proposed equity model suggests that for stop smoking services there is a trade-off to be had between reducing overall prevalence (maximising efficiency) and reducing (or preventing the exacerbation of) socioeconomic inequalities in smoking. We use the example of a stop smoking service in the North East of England to show how the framework can be applied in practice, given existing records kept by SSS in England and the available information on smoking prevalence at small area level.

It has been argued that SSS have a limited impact on smoking prevalence [16]. However, SSS have also been shown to be highly cost-effective compared to other life enhancing interventions [17,18], and SSS will continue to be an attractive use of scarce public health spending. There is therefore a strong practical argument for building an equity element into the evaluation of SSS, in order to avoid the danger highlighted by the Secretary of State of Health, that this spending may exacerbate socioeconomic inequalities in health.

## An equity model of stop smoking services

Within a population covered by a smoking cessation service it will be usual to find socioeconomic inequalities in smoking, whereby the most disadvantaged groups have higher smoking prevalence compared to more affluent groups. If smoking cessation services achieve the same quit rate per smoker for all population groups within the overall population, then it will have no impact on inequalities in smoking prevalence between groups. In order to achieve the same quit rate across all socioeconomic groups it will be necessary for rates of access to SSS to be higher for more disadvantaged groups. This is because clients from more disadvantaged groups are less successful at quitting than clients from affluent groups.

**Figure 1:**
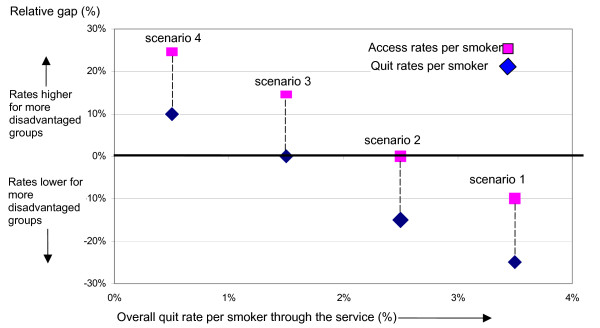
**Equity model of stop smoking services (per smoker)**. The Y axis shows, for quit and access rates, the relative gaps (magnitudes of inequality) between smokers in disadvantaged and affluent wards. The X axis shows overall quit rate for all wards within a stop smoking service.

The model of SSS presented in figure 1 applies to a population covered by a SSS (e.g. local authority or primary care trust). Along the X axis, from left to right, is the percent of smokers in the population as a whole quitting through the SSS. The Y axis depicts the magnitude of inequality in quit rates between disadvantaged and affluent groups within a SSS.

Any overall quit rate per smoker achieved on the X axis will be associated with some degree of socioeconomic inequality in quit rates on the Y axis. The socioeconomic inequality in quit rates between groups may be measured as a relative gap. The methodology used to estimate the relative gaps in this paper is based on the calculation of the slope index of inequality (SII) across wards as described by Low and Low elsewhere [19,20]. Put simply the relative gap is the difference in quit/access rates for smokers in the most deprived compared with those in the most affluent wards, expressed as a percentage of the overall quit rate. For example, if the overall quit rate per smoker is 2%, this may be made up from a quit rate per smoker of 1% in the most affluent wards and a quit rate of 3% per smoker from the most disadvantaged wards. In this case the gap in quit rates between the socioeconomic groups is 2 and the relative gap is 100% (2/2%*100). This would be a positive gap as more disadvantaged wards have the higher quit rates. If the more disadvantaged wards had lower quit rates per smoker then the relative gap would be negative. If there is no difference in quit rates between affluent and disadvantaged wards the relative gap is zero. The relative gap, measured as a point estimate on the Y axis, assesses the direction and magnitude of the socioeconomic inequality in quit/access rates across wards covered by a SSS.

For any SSS, the relative gap in quit rates (diamond marker) will be associated with a relative gap in access rates (square marker), calculated in the same way, on the same scale. For illustrative purposes we have shown the differences in relative gaps in quit and access rates to be the same across all scenarios, although this is may not necessarily be the case in practice.

However, it will always be the case that the diamond marker (relative gap in quit rate) will be below the associated square marker (relative gap in access rate), because clients accessing the service from disadvantaged wards are generally less successful at quitting than those from affluent wards. This means that given, for example, equality of access, i.e. the proportion of smokers in disadvantaged and affluent wards is the same, then quit rates will be lower in more disadvantaged wards.

### Scenario 2 – equity of access

Scenario 2 in figure 1 depicts the situation just described. The quit rate per smoker for the overall population is 2.5%. The proportion of smokers in each socioeconomic group who become clients of the smoking cessation service is the same. In other words there is zero socioeconomic inequality in the number of clients per smoker accessing the service. However, because clients from disadvantaged groups are less successful at quitting, disadvantaged groups have a lower quit rate per smoker. The result is a negative socioeconomic inequality in quit rates per smoker. Because quit rates per smoker are lower in the more disadvantaged groups, the gap in smoking prevalence between disadvantaged and affluent groups will widen.

### Scenario 3 – equity of outcome

In order to ensure that stop smoking services do not contribute to widening inequalities in smoking prevalence between disadvantaged and affluent groups, it is necessary to operate according to scenario 3. Here in disadvantaged groups the proportion of smokers who become clients of the smoking cessation service is higher than for affluent groups. In other words there is positive socioeconomic inequality in the number of clients per smoker accessing the service. But this positive socioeconomic inequality in access is associated with a zero socioeconomic inequality in quit rates per smoker, as clients from more deprived areas are less successful at quitting. In this case quit rates per smoker are the same across socioeconomic groups and the impact on socioeconomic inequalities in smoking prevalence is neutral.

### Scenario 4 – reduction in health inequalities

If stop smoking services are to contribute to reducing inequality in smoking prevalence among the populations they serve, they need to operate according to scenario 4. Here, in disadvantaged groups the proportion of smokers who become clients of the smoking cessation service is higher than for affluent groups. In other words there is positive socioeconomic inequality in the number of clients per smoker accessing the service. This positive socioeconomic inequality in access is of a sufficient magnitude to be associated with a positive socioeconomic inequality in quit rates per smoker. Under this scenario the stop smoking service will contribute to a narrowing of socioeconomic inequalities in smoking prevalence.

### Scenario 1 – maximisation of health improvement

At scenario 1, the proportion of smokers who become clients of the smoking cessation service is lower for disadvantaged groups than affluent ones. This negative socioeconomic inequality in access is associated with an even greater negative inequality in quit rates per smoker, as clients from more deprived areas are less successful at quitting. Suppose there is a fixed capacity of SSS in terms of number of clients it can accommodate. Then, as the proportion of these clients coming from disadvantaged groups falls, the overall number of clients quitting through the service will increase. This scenario therefore maximises the numbers of quitters and thence maximises the impact of the SSS on health improvement.

The above model is a depiction of the mechanics of the relationships between access rates, quit rates, overall reduction in smoking prevalence and reduction in inequalities in smoking prevalence and hence reductions in health inequalities. These relationships follow from the general finding that for those accessing the service, there is a decrease in the likelihood of quitting with increasing deprivation. The clear implication is that there are trade-offs to be had in terms of objectives. The efficiency objective of maximising the impact on prevalence and health improvement must be sacrificed in progressively greater measure to achieve the equity objectives of equal access for equal need, equal outcome for equal need and reduction in inequality in smoking prevalence.

Current SSS targets in local delivery plans are based on numbers of quitters. This only gives a one dimensional view of performance. SSS that are most successful in terms of overall reduction in smoking prevalence may be least successful in reducing socioeconomic inequalities in prevalence.

## Model adaptation to fit data restrictions

Records kept by SSS in England contain information on the numbers accessing the service and successfully quitting. In order to build the equality dimension into performance assessment two other types of information are needed.

First it is necessary to know how those accessing the service and successfully quitting are distributed between socioeconomic groups. SSS in England do not routinely collect socioeconomic data. Information on eligibility for free prescriptions is known and it has been suggested that this could serve as a proxy for socioeconomic group. However we rejected this proxy indicator because, within the 50% of the population who are exempt from prescription charges, many will be from affluent groups, such as pregnant women and pensioners.

We follow NEPHO [11] and select postcode as a best available indicator of socioeconomic position. The client's postcode is normally recorded and this can be used to determine the distribution of clients across socioeconomic groups depending on their ward of residence and the associated deprivation score of that ward, based on the Index of Multiple Deprivation (ID 2004).

Second, because the analysis is conducted in terms of quit rates per smoker, we need to know not only how clients are distributed across socioeconomic groups, but also how smokers are distributed across these groups. The Neighbourhood Statistics team have developed synthetic smoking prevalence estimates at ward level. These are estimates based on the demographic and social characteristics of the area and cannot be used to monitor smoking prevalence at individual ward level over time. They cannot therefore be used to estimate quitters per smoker at different periods of time.

**Figure 2:**
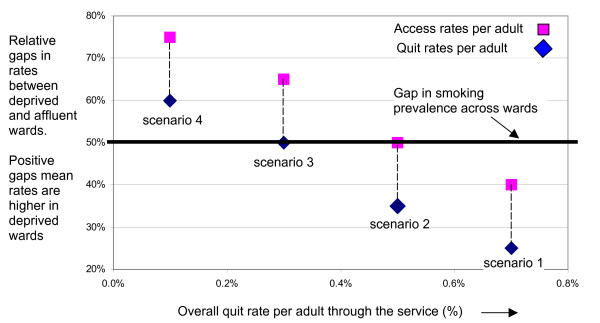
Adapted model of stop smoking services (per adult).

However we suggest the synthetic smoking prevalence data can provide a reasonable indicator of the size of the gap in smoking prevalence per adult across the wards covered by a stop smoking service. This prevalence gap estimate can be superimposed onto an analysis of access and quit rates per adult, instead of per smoker. This is illustrated in figure 2 and the interpretation of the scenarios is exactly as explained for figure 1.

In figure 1 the benchmark for assessing equity was a zero gap in access and quit rates per smoker between disadvantaged and affluent wards. In figure 2 the benchmark for assessing equity is a 50% gap in rates per adult, which is the size of the gap in smoking prevalence between disadvantaged and affluent wards.

The change in the benchmark for assessing equity of access and quitting from zero with a per smoker analysis to the gap in smoking prevalence (e.g. 50%) in the per adult analysis is merely a mathematical function of the change in denominator. This is shown in the simple numerical example in Table 1.

**Table 1:** Example to show relationships between quitting rates per smoker and per adult and smoking prevalence

	No. adults	No. smokers	No. quitters
Deprived group	100	25	5
Affluent group	100	15	3

In the example above, the quit rate per smoker is the same for both groups (20%). This means there is a zero gap in the quit rate. However the quit rate per adult is 5% for the deprived group and 3% for the affluent group. The relative gap is +50% (absolute gap in quit rates of 2%, divided by overall quit rate of 4%). This relative gap in quit rate of +50% is the same as the gap in smoking prevalence (absolute gap in smoking prevalence of 10%, divided by overall prevalence of 20%). The equality of quit rate condition is met, since the gap in quit rate per adult is the same as the gap in smoking prevalence for adults.

## Application to Derwentside PCT

Between 2001/02 and 2005/06 there have been changes in how the SSS in Derwentside Primary Care Trust is organised. The Derwentside Stop Smoking Service was launched in January 2001 with the service initially fed predominantly by GP referrals, either to group support or to a 1:1 session. Under this model, the service success rate was good but the number of people seen was well below the requirement of the Local Delivery Plan target. In 2004, the service was expanded and moved to a model of delivery focussed around intermediate advisors, paying GP practices and pharmacists for their input. The intermediate advisors, including health visitors, practice nurses and pharmacists, were trained to deliver support to quitters, a role which was in addition to their other responsibilities. The expansion included the training of most practice nurses to become smoking cessation advisors. In addition six health visitors have been trained as level 2 advisors. Table 2 shows the impact of this service expansion in terms of numbers accessing and successfully quitting.

**Table 2:** Numbers accessing and quitting 2001/02 to 2005/06

**Year**	**16+ population**	**Number accessing**	**Number quitting**	**LDP quitting target**
2001/02	65300	393	246	None set
2002/03	66000	324	194	None set
2003/04	66100	1,149	591	515
2004/05	67600	1,733	671	515
2005/06	67600	1,895	608	605

In order to assess the equity effects of these changes, stop smoking data for the two periods 2001/02 and 2004/05 were analysed. These two years were selected as they represented the earliest and latest years for which full data were available at ward level. They also represent years before and after the change in the SSS delivery model. For each period relative gaps between the most affluent and deprived wards across all 22 wards in Derwentside have been calculated for access rates and quit rates per adult. The 22 wards in Derwentside were ranked from most deprived to most affluent on the basis of the income domain of the Index of Multiple Deprivation 2004, aggregated from super output area (SOA) to ward level. The size of the absolute gap in rates across all wards arranged from most affluent to most deprived was calculated using the slope index of inequality (SII). The relative gap was then estimated as the absolute gap divided by the average rate across all wards[20]. The data used for these calculations are provided in additional file 1.

**Figure 3:**
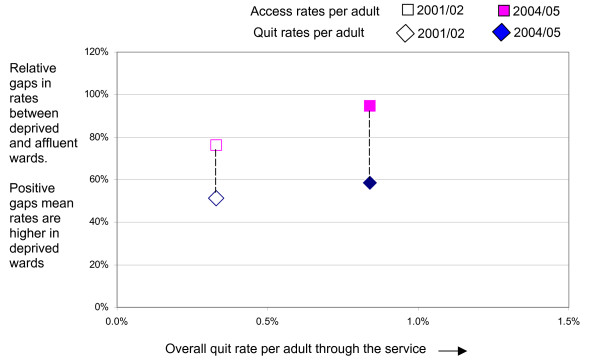
Direction of travel analysis 2001/02-2004/05: access and quit rates per adult for Derwentside PCT.

The results are shown in figure 3. Between the two periods two advances in performance have been made. First service changes and expansion have resulted in the percent of adults in Derwentside quitting through the SSS increasing from 0.33% to 0.8%. At the same time there has been a shift in the distribution of additional resources into the service in favour of the more deprived areas of Derwentside.

It can be seen that service expansion has resulted in the desired direction of travel. That is, to the right (increasing overall quit rate) and upwards, a shift in the distribution of quitters in favour of deprived wards, where the prevalence/need is higher.

Whether this shift has been sufficient to meet equity objectives or to ensure that the service is contributing to the reduction of inequality in the prevalence of smoking cannot be known until the smoking prevalence gap is incorporated into the analysis. The smoking prevalence gap across wards in Derwentside has been calculated in the same way as for access and quit rates. The relative gap between affluent and deprived wards is 71%, based on synthetic estimates of prevalence using 2004/05 data.

This prevalence gap estimate can be compared with an alternative estimate across Derwentside using general practice data. In 2005 smoking prevalence data were collected from general practices. In these practices 68% of patients aged over 16 had had their smoking status recorded within the last three years. The estimated prevalence of smoking across Derwentside from this source was 29.4%, which compares with 31% for the synthetic estimate. The Health Survey for England estimated that 28% men and 30% of women in the North East smoke [21]and a local market research survey indicated that 33% of adults in Derwentside smoke [22]. Thus the estimated smoking prevalence from general practice records is in line with other estimates. The general practice data also enabled prevalence to be estimated at ward level, as the postcodes of patients having their smoking status checked were known. These general practice based ward level smoking prevalence rates generated a relative gap of 69% [13].

The prevalence gap estimate based on synthetic data (71%) is very close to that based on general practice data (69%). This suggests that synthetic data may provide a reasonable estimate of prevalence gaps across wards where other information is not available. In this paper we use the synthetic estimate.

**Figure 4:**
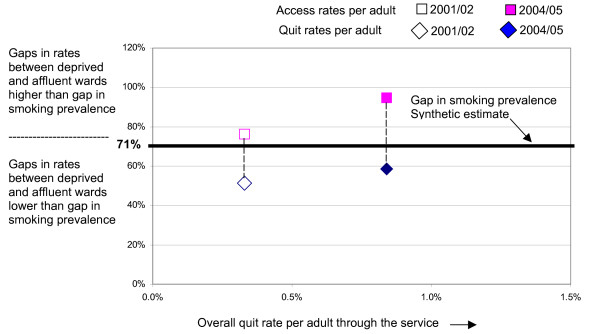
Equity and inequality analysis: access and quit rates per adult for Derwentside PCT.

In figure 4 this estimate of the relative gap in the rate of smoking per adult across wards in Derwentside has been superimposed onto figure 3 as a bold horizontal line. This provides the benchmark, which enables equity of access and quitting to be assessed. It also enables the question of whether stop smoking services are contributing to reducing inequalities in smoking prevalence to be answered.

The analysis in figure 4 suggests that the Derwentside Stop Smoking Service is operating at a position between scenario 2 (equity of access) and scenario 3 (equity of outcome). Between 2001/02 and 2004/05 there has been some movement towards scenario 3, but equity of outcome is not yet being achieved. This equity assessment would have been no different if the smoking prevalence gap used was that based on general practice data (69%).

The fact that the gap in quit rates per adult between affluent and deprived areas is lower than the gap in smoking prevalence means that the Stop Smoking Service in Derwentside is not contributing to a reduction of inequality in smoking prevalence between deprived and affluent areas.

## Discussion

How should SSS be assessed? Individual stop smoking services set targets in terms of numbers quitting per year. These targets are aimed at reducing overall prevalence in the local population. But there are also expectations that SSS can and should contribute to the reduction in health inequalities by reducing smoking prevalence gaps between socioeconomic groups. For example a North East Public Health Observatory study examined whether regional SSS are effective in reducing health inequalities [11]. One of the six specific service priorities in the NHS Operating Framework for 2006/07 is the reduction of health inequalities and the initial focus here is to be on implementing smoking cessation interventions and tracking their performance [12].

It seems important therefore to be able to assess SSS on both the dimensions of overall prevalence reduction and on changes in the distribution of prevalence between disadvantaged and affluent groups. We have proposed an equity model, which enables performance in terms of both these dimensions to be assessed at the same time.

The full application of this model to SSS requires smoking prevalence data to be available by area or socioeconomic group. Although these data are only readily available at small area level in synthetic form, we have suggested a way in which the synthetic estimates may be used. However it remains the case that synthetic estimates of smoking prevalence are based on socioeconomic deprivation scores and will therefore be correlated with access and quit gap estimates, also based on socioeconomic deprivation scores. We have shown that direct estimates of smoking prevalence can be used as an alternative, with, for Derwentside, very little change in the results.

Even if it is considered that estimates of the magnitudes of gaps in smoking prevalence based on synthetic data are not reliable and other estimates are not available, the model can be applied in a partial way to track performance in both dimensions over time. Most SSS will have accumulated data over a number of years on the number of quitters by postcode. These can be analysed for two or more periods to examine the direction of travel over time as in figure 3.

A question arises as to the level at which assessments should be made. The NEPHO study, across 11 separate SSS, indicated that over the whole of the North East, quit rates per smoker were higher in more deprived areas and that the service was therefore decreasing the socioeconomic inequality in smoking prevalence [11]. This does not mean, however, that each of the 11 services is contributing to the reduction of inequalities in smoking prevalence among the populations they serve. A region-wide analysis across many stop smoking services reveals the broad picture. However, for planning and performance management, it is important to undertake assessments at the level of individual stop smoking services.

Milne [16] has argued that stop smoking interventions are not the best way to reduce smoking prevalence and that comprehensive restrictions on smoking in all workplaces works better. However he also acknowledges that both are needed and, to reduce health inequalities, deprived areas need more of both. We agree that the distribution of capacity between more deprived and more affluent areas is as important as the development of overall capacity. Unless the distributional and equity impact of SSS are assessed, it will not be possible to know whether services are contributing to the reduction of inequalities in smoking prevalence or not, and the danger highlighted by the Secretary of Health will go undetected and unaddressed.

## Competing interests

The author(s) declare that they have no competing interests.

## Authors' contributions

AlL conceived of the analytical model and wrote the first draft. LU acquired the data and undertook the analysis. AnL initiated the study and assisted with drafting the manuscript. IM facilitated the collection of the data and implemented the change in model of SSS delivery. All authors read and approved the final manuscript.

## Pre-publication history

The pre-publication history for this paper can be accessed here:



## Supplementary Material

Additional file 1 Additional file: Dataset used to generate Figures 3 and 4. The data provided represent the information used to calculate the relative gaps (relative index of inequality – RII) in access and quit rates per adult, which are depicted in Figures 3 and 4 Click here for file
