# Supplementary material for: Avoiding the danger that stop smoking services may exacerbate health inequalities: building equity into performance assessment
Source: BMC Public Health. 2007 Aug 9;7:198. doi: 10.1186/1471-2458-7-198 (PMC1964765; doi:10.1186/1471-2458-7-198)
Supplement: Additional file 1 — Additional file: Dataset used to generate Figures 3 and 4. The data provided represent the information used to calculate the relative gaps (relative index of inequality – RII) in access and quit rates per adult, which are depicted in Figures 3 and 4 [file 1471-2458-7-198-S1.doc]

**Additional file: Dataset used to generate Figures 3 and 4**

|  | IMD 2004 income deprivation score | 16+ registered population  2005 | Synthetic estimate of smoking prevalence | % Population accessing service  2001/2 | % Population accessing service  2004/5 | % Population quitting at 4 weeks  2001/2 | % Population quitting at 4 weeks  2004/5 |
| --- | --- | --- | --- | --- | --- | --- | --- |
| Castleside | 0.0738 | 1300 | 20.4 | - | 1.20% | - | 0.45% |
| Lanchester | 0.0791 | 3400 | 19.7 | 0.23% | 1.66% | - | 0.52% |
| Benfieldside | 0.0911 | 3800 | 20.5 | 0.50% | 1.56% | 0.32% | 0.72% |
| Burnopfield | 0.1018 | 3700 | 21.9 | 0.38% | 2.09% | 0.27% | 0.92% |
| Consett East | 0.1155 | 1700 | 35.7 | 0.48% | 1.27% | - | 0.66% |
| Ebchester and Medomsley | 0.1415 | 3900 | 20.1 | 0.64% | 2.22% | 0.41% | 0.84% |
| Consett North | 0.1443 | 4200 | 29.6 | 0.59% | 1.97% | 0.29% | 0.74% |
| Tanfield | 0.1486 | 3700 | 29.3 | 0.46% | 1.50% | 0.16% | 0.64% |
| Esh | 0.1524 | 1700 | 29.6 | 0.36% | - | - | - |
| Cornsay | 0.1636 | 200 | 32.4 | - | - | - | - |
| Blackhill | 0.1762 | 4100 | 32.8 | 0.88% | 1.85% | 0.58% | 0.63% |
| Havannah | 0.1763 | 4000 | 27.2 | 0.52% | 1.85% | 0.30% | 0.57% |
| Delves Lane | 0.1827 | 4200 | 34.2 | 0.67% | 2.21% | 0.39% | 0.72% |
| Dipton | 0.1963 | 2300 | 29 | 0.35% | 1.87% | 0.13% | 0.57% |
| South Moor | 0.2046 | 3800 | 35 | 0.63% | 2.69% | 0.37% | 0.79% |
| Leadgate | 0.2210 | 3800 | 34.2 | 1.20% | 4.56% | 0.59% | 1.33% |
| Annfield Plain | 0.2268 | 3800 | 34.9 | 0.68% | 3.28% | 0.31% | 0.68% |
| Catchgate | 0.2280 | 2500 | 33.9 | 1.02% | 3.49% | 0.24% | 1.22% |
| Burnhope | 0.2357 | 900 | 31.6 | 1.17% | 2.13% | 0.64% | - |
| Stanley Hall | 0.2435 | 4000 | 37 | 0.72% | 2.17% | 0.32% | 0.84% |
| Consett South | 0.3167 | 2600 | 40.5 | 0.77% | 2.90% | 0.27% | 1.32% |
| Craghead and South Stanley | 0.3374 | 4000 | 46.7 | 0.93% | 4.07% | 0.48% | 1.18% |
| Derwentside SSS | 0.1819 | 67600 | 30.7 | 0.64% | 2.34% | 0.33% | 0.79% |
